# Complex regional pain syndrome: diagnostic challenges and favorable response to prednisolone

**DOI:** 10.1186/s12891-024-07333-0

**Published:** 2024-04-10

**Authors:** Jimmy Olomi, Victoria Munthali

**Affiliations:** 1 University of Dar es salaam (UDSM), P.O. Box 608, Mbeya, Tanzania; 2 Muhimbili Orthopedic Institute (MOI), P.O. Box 65474, Dar es salaam, Tanzania; 3 Mbeya zonal referral hospital (MZRH), P.O. Box 419, Mbeya, Tanzania

**Keywords:** CRPS, Complex regional pain syndrome, Chronic pain, Hand swelling, Unilateral hand swelling, Limb swelling

## Abstract

Complex regional pain syndrome (CRPS), characterized by severe and disproportionate pain, is a rare and debilitating condition. Due to its rarity, evidence-based treatment guidelines remain limited, creating a challenge for clinicians. We present the case of a 20-year-old female with CRPS type 1 of the right hand. Her pain, initially triggered by a minor trauma, had persisted for three months. The patient demonstrated severe pain, swelling, hyperesthesia, and restricted range of motion. Despite multiple hospital visits, her symptoms did not improve until she was diagnosed with CRPS and treated with oral prednisolone. A dosage of 40 mg daily led to a dramatic response within 10 days. Our report emphasizes the importance of recognizing CRPS and highlights the potential of prednisolone as a treatment option, particularly in resource-limited settings, where more specialized interventions may be unavailable. Further research is essential to establish a stronger evidence base for the use of steroids in CRPS management.

## Introduction

Complex regional pain syndrome (CRPS) was a name adopted by the International Association for the Study of Pain (IASP) in 1994 to describe a chronic and disabling condition that mainly affects extremities.

Over the years, its definition and diagnosis criteria have been changing; however, the widely accepted definition is that “*CRPS describes an array of painful conditions that are characterized by a continuing (spontaneous and/or evoked) regional pain that is seemingly disproportionate in time or degree to the usual course of any known trauma or other lesion”.* The pain is regional (not in a specific nerve territory or dermatome) and usually has a distal predominance of abnormal sensory, motor, sudomotor, vasomotor, and/or trophic findings. The syndrome shows variable progression over time [1].

CRPS is a rare entity with an incidence of 5.4–26.2 per 100 000 person years [2], affecting more females (4:1) and upper limbs [3].

In this paper, we report a patient who responded well to steroid therapy and hope to remind clinicians of the disease and support the use of steroids in the case of limited resources for the use of interventional therapies.

### Case report

We describe a case of a 20-year-old female who sought medical attention due to severe and persistent right-hand pain lasting three months. The pain was sudden in onset, characterized by intensity, burning sensation, and occasional pins and needles. It worsened with limb elevation and was triggered by superficial touch but alleviated by ice application and meloxicam. The pain was accompanied by persistent swelling that initially appeared at the wrist and progressed to involve the entire hand. Notably, this was the third episode of similar symptoms in the same hand over two years. The patient had previously experienced a milder episode lasting less than three weeks, following a history of school-related trauma. During the current episode, she had sought treatment in different hospitals, receiving antibiotics and analgesics with no improvement. A biopsy was performed one month before her visit to our clinic. Her medical history was largely unremarkable, and she was a college student with no history of alcohol or cigarette use.

**Fig. 1:**
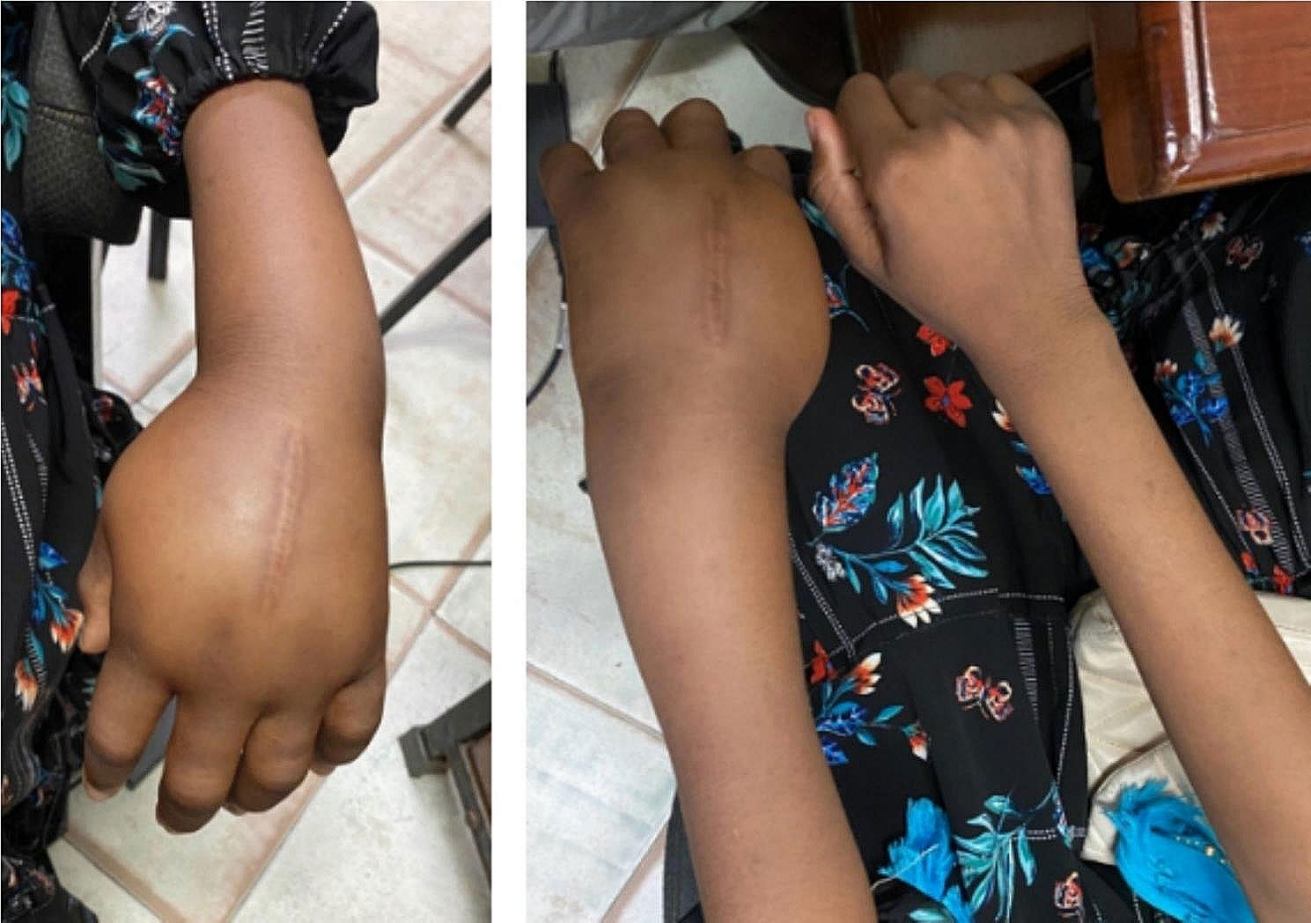
Pictures of the hand on the day of presentation

Physical examination revealed significant swelling in the hand, wrist, and distal forearm, extending approximately 10 cm from the wrist joint. The affected area displayed a longitudinal biopsy scar, darker skin, and hair loss (Fig. 1). Hyperesthesia was noted without a specific dermatomal distribution. Pitting edema was present, and both the Stemmer and Tinel signs were negative (Phalen’s sign was not elicited). The capillary refill was less than 2 s, and the affected hand had a lower temperature than the contralateral hand. Pain limited the range of motion in the finger and wrist joints, with a passive flexion-extension arc of only 30 degrees at the wrist.

**Fig. 2:**
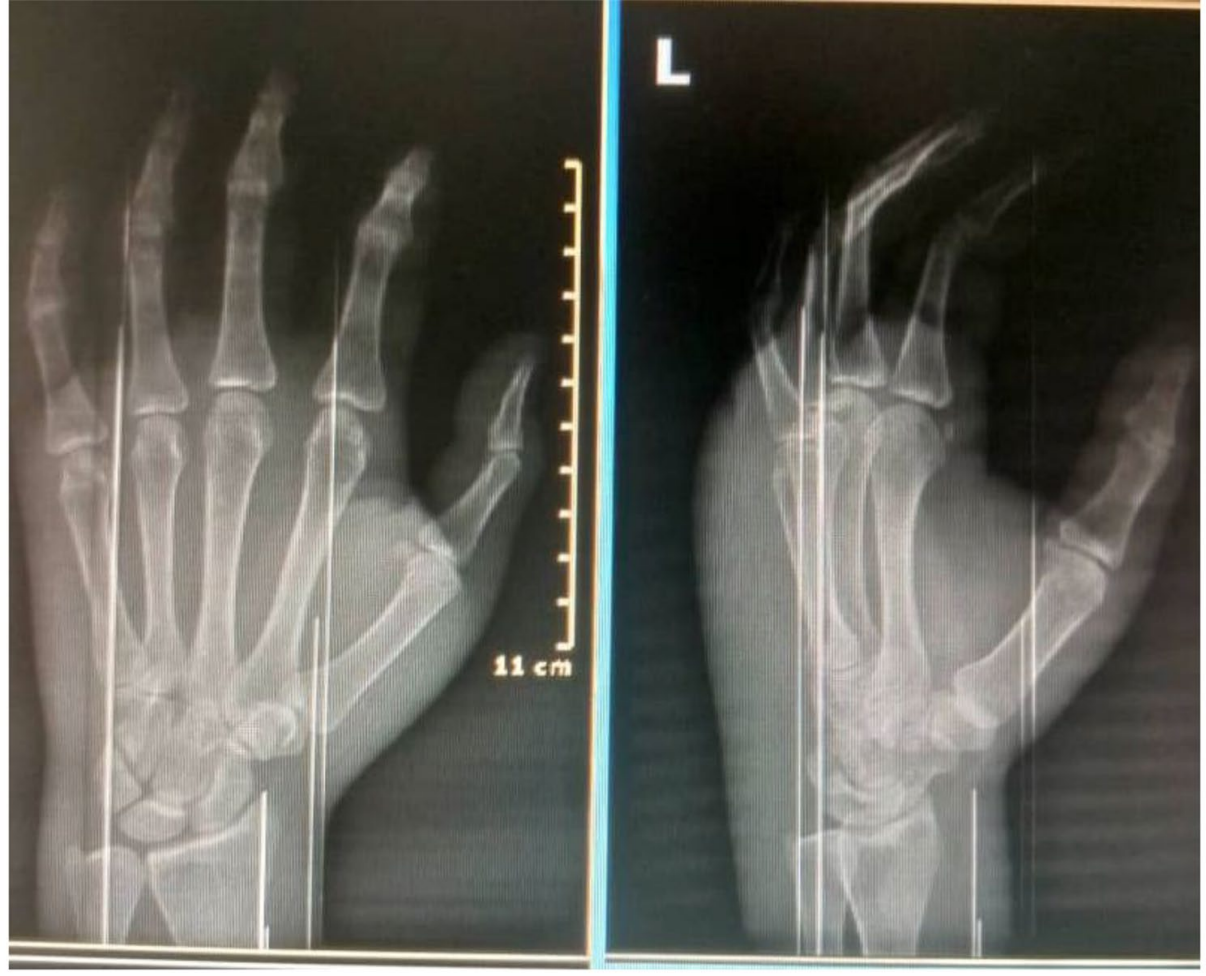
Xray image of the left hand

**Fig. 3:**
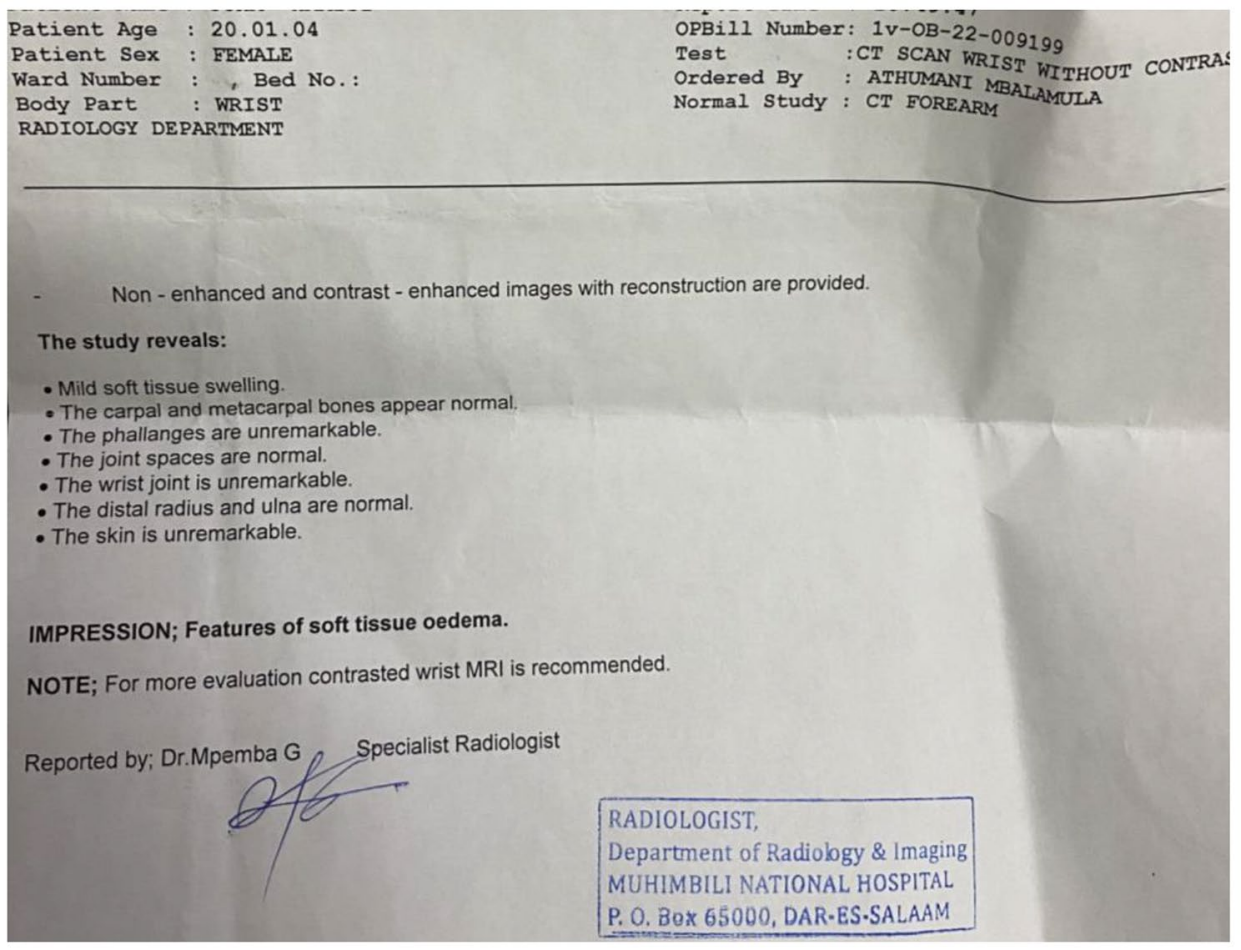
A CT scan report

**Fig. 4:**
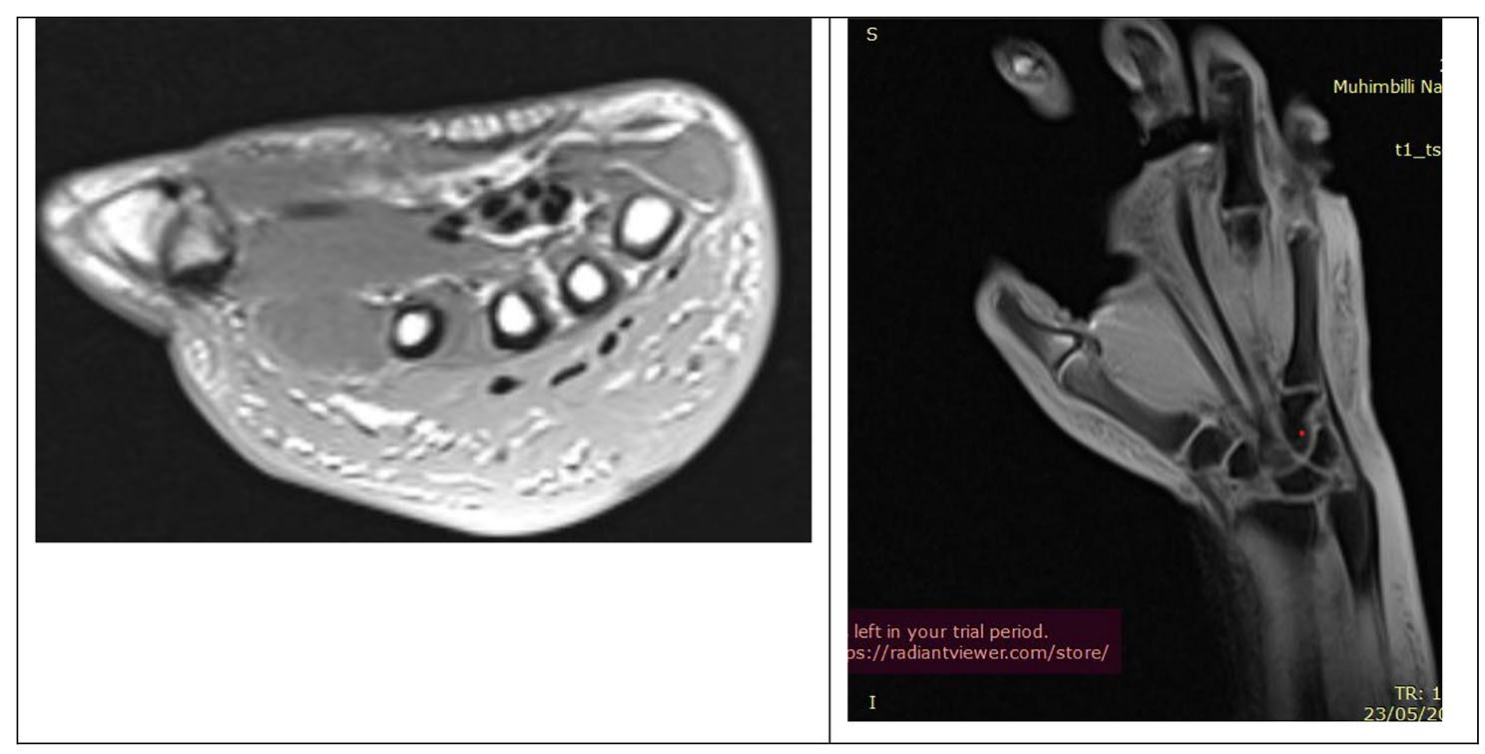
MRI images of the hand: axial and coronal cuts

Laboratory investigations, including a complete blood count, C-reactive protein (CRP), and erythrocyte sedimentation rate (ESR), all fell within the normal range. Hand radiographs (Fig. 2) revealed significant soft tissue shadows and signs of osteopenia, without other identifiable pathologies. A CT angiogram (Fig. 3) yielded normal results, MRI (Fig. 4) showed edematous tissue in the hand and wrist. And Previously performed biopsyindicated normal tissue.

In alignment with the Budapest criteria, we diagnosed her with complex regional pain syndrome (CRPS) type 1 of the left hand.

**Fig. 5:**
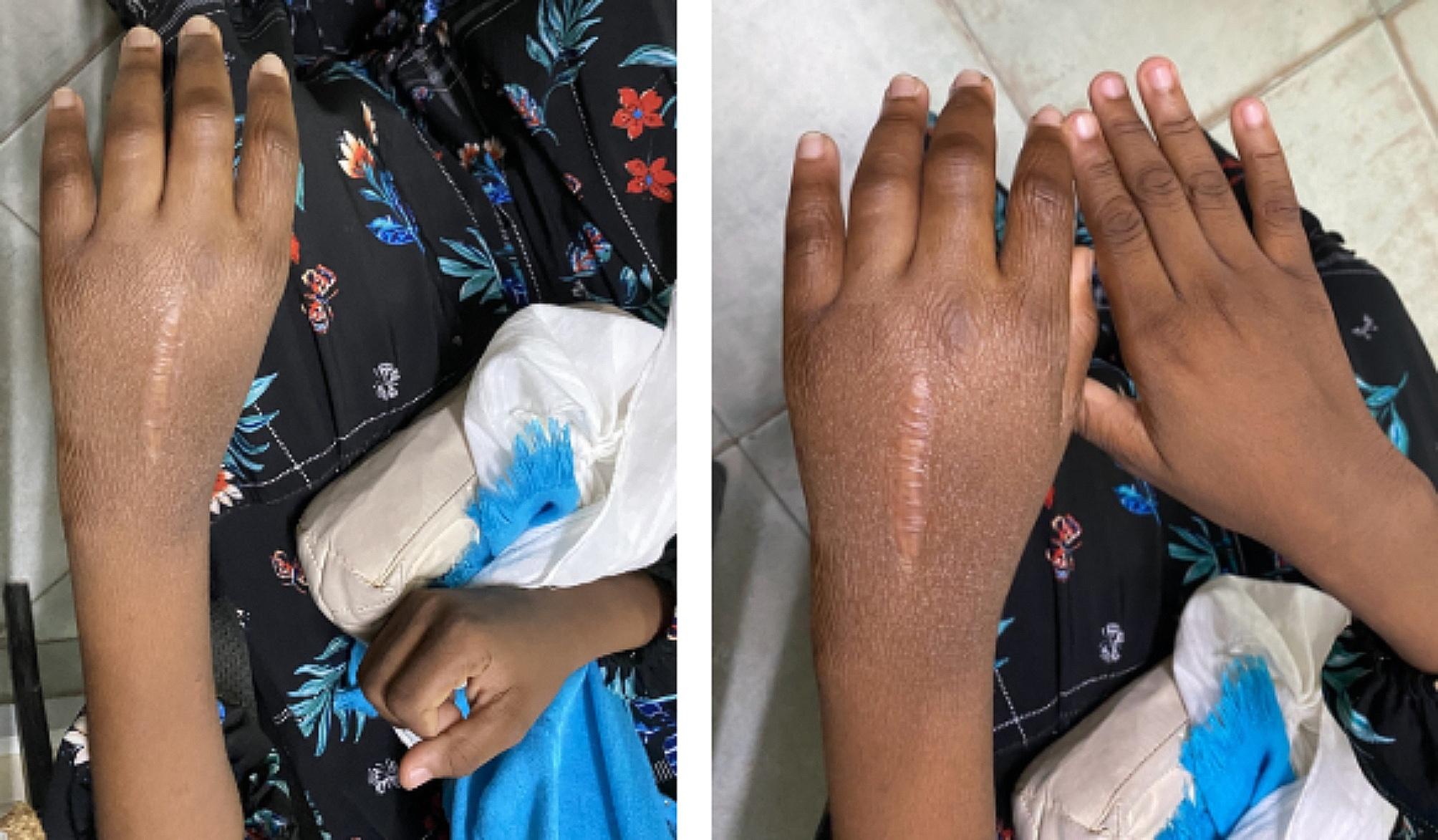
Pictures of the hand 7 days on prednisolone (left picture the patient instructed to make a fist)

**Fig. 6:**
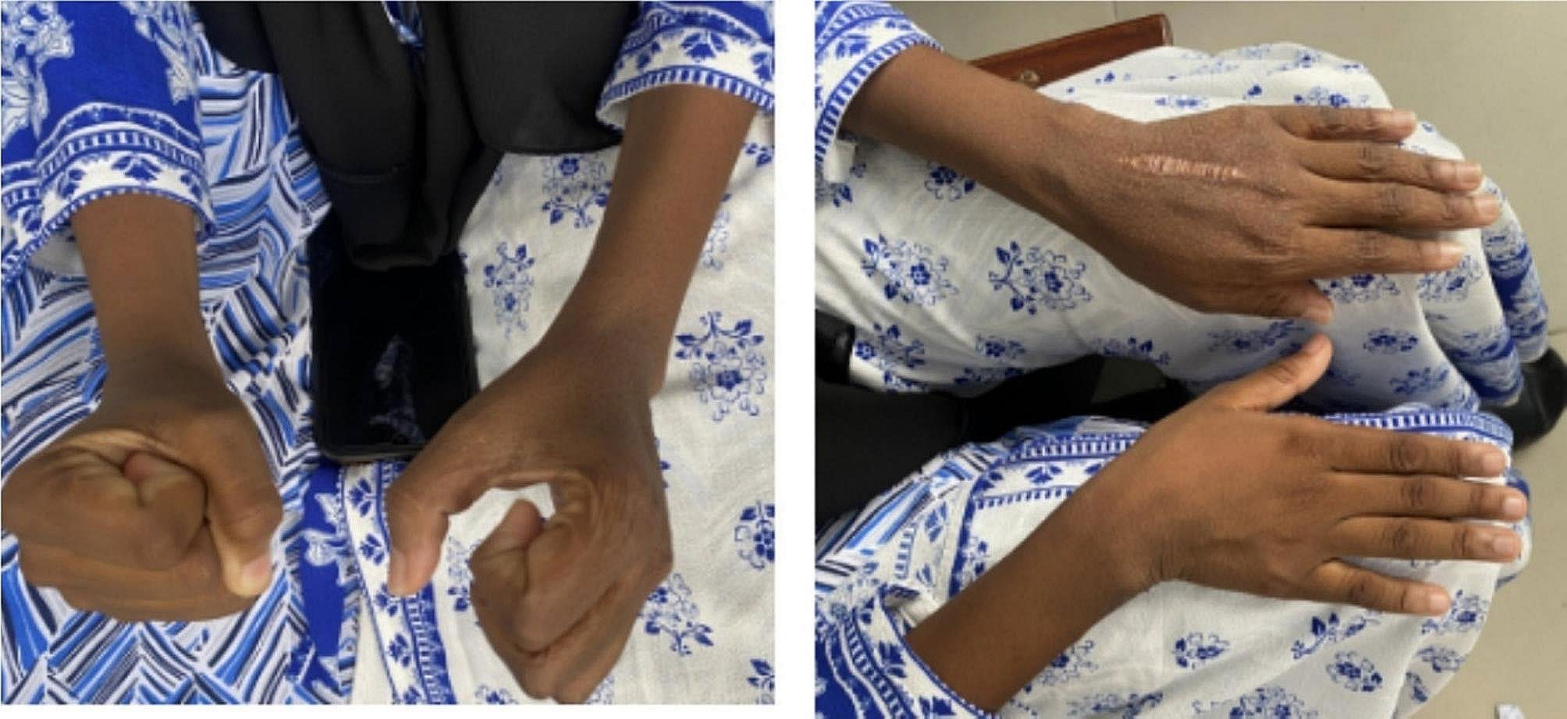
Pictures of hands 12 days on prednisolone (left picture patient instructed to make a fist)

Treatment with oral prednisolone at a dosage of 40 mg daily resulted in a dramatic response after 7 days (Fig. 5), and after 12 days on prednisolone no swelling was noted and the pain had significantly subsided although some residual hand grip weakness remained (Fig. 6). Prednisolone was tapered over six weeks. During the six-month follow-up period, the patient was kept on physical therapy and remained free from pain and was able to return to college with full function of the hand.

## Discussion

Complex Regional Pain Syndrome (CRPS) can be categorized into two main types based on whether identifiable nerve damage is present: CRPS type 1, the more common subtype, lacks identified major nerve injury, while CRPS type 2 with identified major nerve damage [3, 4]. This subtyping was later adopted as the formal subtype in the 2012 IASP CRPS criteria and correspond to the previous names: reflex sympathetic dystrophy (RSD) for CRPS-type and causalgia for CRPS-type 2 [2, 3].

Recently two more subtypes have been identified including CRPS-NOS (not otherwise specified) which refers to cases where individuals exhibit symptoms partially meeting CRPS criteria, yet cannot be attributed to any other specific condition and CRPS with remission of some features (CRSF) where certain characteristics of CRPS are in remission [5].

Phenotypically two types have been described including inflammato- ry or warm CRPS, and chronic or cold CRPS [5], where the majority are usually the cold type [6] like the patient we describe in this report.

### Pathophysiology and etiology

While the precise pathophysiology of complex regional pain syndrome remains unclear, it is through the observation of patients’ presentations and the examination of abnormalities found in individuals with this condition that several mechanisms have been postulated [2, 7].

These include nervous system sensitization, autonomic nervous system dysfunction and inflammation.

Both the peripheral and central nervous systems become sensitized in complex regional pain syndrome (CRPS). Peripheral nervous system (PNS) sensitization is driven by the presence of pro-inflammatory cytokines, such as TNF-alpha and prostaglandin E2, which are locally released after a trauma or inciting event. These cytokines reduce the depolarization threshold, making nerves more excitable [8].

Central nervous system (CNS) sensitization, on the other hand, results from increased synaptic nociceptive firing in the dorsal horn of the spinal cord, which leads to the production of neurotransmitters such as substance P and glutamate, which lower the threshold for the nervous system’s response to mechanical and thermal stimuli [8].

As a consequence of this sensitization, patients with CRPS often experience allodynia (pain from typically nonpainful stimuli) and hyperesthesia (increased sensitivity to sensory stimuli).

There are also structural changes observed in the peripheral nervous system. In a recent case report of a patient with refractory CRPS, an analysis of nerve tissue from the amputated limb revealed selective degeneration of Aα fibers (motor and proprioception), while smaller Aδ fibers (pain and temperature sensation) were spared [9]. This can also explain the allodynia and hyperalgesia observed in CRPS patients.

Inflammation, an expected tissue response to trauma, is associated with amplified and persistent innate immunity activation in individuals with CRPS. This results in a sustained release of proinflammatory cytokines, which explains the redness, swelling, pain, and warmth that is characteristic of the acute phase of CRPS. These cytokines also activate osteoclasts and osteoblasts and increase bone turnover, and this is evident in local osteopenia, a common presentation of patients with CRPS.

An autoimmune and autoinflammatory hypothesis has been proposed, albeit with limited supporting evidence. Studies on CRPS patients have revealed elevated levels of autoantibodies targeting β2-adrenergic receptors and m2-acetylcholine receptors. However, the clinical significance of these findings remains unproven. Notably, attempts to treat CRPS through sympathetic nervous system blockade have not demonstrated significant efficacy [10]. 

### Diagnosis

Complex regional pain syndrome presents a significant challenge in both diagnosis and treatment. Because of the rarity of the condition, clinicians do not encounter patients with these presentations and are not familiar with the diagnostic criteria.

The International Association for the Study of Pain (IASP) has dedicated significant efforts to standardizing terminologies and diagnostic criteria through various consensus meetings [1, 11]. Notably, in 2003, a pivotal meeting took place in Budapest, Hungary, led by Harden et al., which resulted in the formulation of the Budapest criteria, as outlined in Table 1 [1]. This has since improved the clinical diagnosis of CRPS [6].

Our patient met all the Budapest criteria; she had continued pain that was disproportionate to the inciting event (caned at school 2 years back), and she reported hyperesthesia and allodynia, asymmetry in skin temperature, hair and color, swelling and edema, and decreased range of motion. There was no other diagnosis to better explain the condition.

There is no diagnostic test for CRPS; however, some modalities are known to support the diagnosis, including.

CRPS can manifest differently among individuals, often requiring various imaging modalities such as MRI, CT, CT Angiography, plain radiographs, ultrasound, and other investigations to rule out alternative explanations for these presentations [12]. In our case, an MRI and biopsy were performed to exclude the possibility of a soft tissue tumor, a CT Angiography was conducted to rule out the presence of venous thrombosis, and X-rays were taken to exclude bone tumors or fractures.

Other modalities that may aid in the diagnosis include thermography (changes of 1^0^ C or more are considered significant [13]), bone scintigraphy and electromyography.

### Treatment

The scarcity of robust evidence supporting treatment for complex regional pain syndrome (CRPS) is underscored in the latest treatment guidelines, including the 5th edition published in 2022. The rarity of this condition poses a significant challenge for conducting randomized clinical trials due to difficulties in patient recruitment [4].

In pain management resource -limited settings, a combination of pharmacotherapy and physical therapy is the most feasible treatment approach for complex regional pain syndrome (CRPS).

Various pharmacological agents have been explored as potential treatments, including nonsteroidal anti-inflammatory drugs (NSAIDs), steroids, tricyclic antidepressants, gabapentin, pregabalin, carbamazepine, opioids, clonidine, nifedipine, alpha-adrenergic antagonists, lidocaine patches, topical capsaicin, calcitonin and bisphosphonates. While these medications have been studied in the context of CRPS management, the evidence supporting their effectiveness remains limited [4].

The effectiveness of prednisolone (steroids) in treating CRPS was assessed in a retrospective cohort study conducted in Canada. Notably, a significant proportion of the participants (97.4%) reported either no pain or minimal pain that enabled them to engage in daily activities after using prednisolone [14]. 

A randomized trial assessing the effectiveness of oral prednisolone in treating CRPS among stoke patients reported a significant improvement of patients receiving prednisolone [14], such improvement was seen in our patient, suggesting that prednisolone may hold promise as a valuable therapy for managing CRPS.

Bisphosphonates, particularly neridronate, have been extensively researched for the treatment of CRPS, and neridronate, currently authorized solely in Italy for CRPS management, shows significant promise [15–17]. 

Although the exact mechanism of action of bisphosphonates remains unclear, it is believed that they suppress macrophage activation, disrupt pro-inflammatory mediators, control Nerve Growth Factor expression, and modulate microenvironmental pH, demonstrating potential analgesic effects. In a Randomized Controlled Trial (RCT) leading to neridronate approval in Italy for CRPS treatment, it outperformed the placebo in improving CRPS symptoms [15, 18]. 

Additionally, specific patient populations with a known risk for developing complex regional pain syndrome, such as elderly women with distal radius fractures, may benefit from the use of vitamin C, as studies have shown it to be superior to a placebo in preventing CRPS [19].

Rehabilitation cannot be overly emphasized in the management of CRPS. This condition is typically linked to muscle dystrophy and joint stiffness resulting from prolonged disuse and immobilization of the affected joints. Although available literature is limited to give strong recommendations for the use of some of the therapies in the treatment of CRPS, Moretti et al. in 2020 evaluated the effectiveness of various physical agents for CRPS Type 1 treatment. They identified Electromagnetic Field Therapy, including pulsed electromagnetic field (PEMF) and bio-electro-magnetic-energy-regulation (BEMER) magneto-therapy, as effective in alleviating CRPS symptoms. Additionally, Transcutaneous Electrical Nerve Stimulation (TENS) emerged as a promising physical therapy approach, supported by positive outcomes in various case series and reports [20]. 

Moreover, the review highlighted other modalities such as Scrambler therapy and Laser therapy, further expanding the spectrum of effective treatments for CRPS Type 1 based on the available evidence [20]. These modalities can be combined with other physical exercises that will improve range of motion across the affected joints, however, overly aggressive or excessive physical therapy may worsen inflammation and contribute to increased pain, edema, and fatigue [4]. 

Other modalities encompass interventional therapies such as Sympathetic Nerve Blocks, IV Regional Nerve Blocks, Somatic Nerve Blocks, Epidural and Plexus Catheter Infusion/Block(s), Neurostimulation, Intrathecal Drug Infusion (e.g., Baclofen or Clonidine), Sympathectomy, and Motor Cortex Stimulation, listed in order of increasing aggressiveness [2, 4].

## Conclusion

Complex regional pain syndrome (CRPS) is a rare and challenging condition. Our case report serves as a reminder to clinicians of the existence of this condition and its potential severity. The successful treatment of our patient with oral prednisolone highlights the possibility of using steroids, particularly in resource-limited settings where interventional therapies are not readily available. This case underscores the need for further research to establish stronger evidence for the use of steroids in managing CRPS, given its complex and poorly understood nature.

**Table 1:** Budapest criteria

1	2	3	4
**Continuing pain, which is disproportionate to any inciting event**	**Must report at least one symptom in three of the four categories**	**Must display at least one sign at time of evaluation in two or more of the four categories**:	**There is no other diagnosis that better explains the signs and symptoms**
	*1.Sensory*: *Reports of hyperesthesia and/or allodynia.*	*1.Sensory*: *hyperalgesia (to pinprick) and/or allodynia.*	
	*2.Vasomotor*: *Reports of temperature asymmetry, skin color changes, skin color asymmetry.*	*2.Vasomotor*: *temperature asymmetry (> 1 °C), skin color changes.*	
	*3.Sudomotor/Edema*: *Reports of edema, sweating changes, sweating asymmetry.*	*3.Sudomotor/Edema*: *edema, sweating changes, sweating asymmetry.*	
	*4.Motor/Trophic*: *Reports of decreased range of motion, motor dysfunction, trophic changes (hair, nail, skin)*	*4.Motor/Trophic*: *decreased range of motion and/or motor dysfunction, trophic changes.*	

To make a clinical diagnosis, all of the criteria must be met

## Data Availability

The data presented in this study are available on request from the corresponding author.
